# Fluoroquinolone preventive therapy for children exposed to MDR-TB

**DOI:** 10.5588/ijtld.21.0443

**Published:** 2022-02-01

**Authors:** T. Gureva, A. Turkova, E. Yablokova, P. Smirnova, O. Sveshnikova, O. Zolotaya, E. Nikishova, E. Heldal, S. Hinderaker, J. A. Seddon, A. Mariandyshev

**Affiliations:** 1 Northern State Medical University, Arkhangelsk, Russian Federation; 2 MRC Clinical Trials Unit at University College London, Institute of Clinical Trials & Methodology, London, UK; 3 Arkhangelsk Clinical Antituberculosis Dispensary, Arkhangelsk, Russian Federation; 4 Heart and Lung Patient Organization International, Oslo, Norway; 5 University of Bergen, Bergen, Norway; 6 Imperial College London, London, UK; 7 Northern Arctic Federal University, Arkhangelsk, Russian Federation

Dear Editor,

Modelling studies suggest that ~30,000 children develop multidrug-resistant TB (MDR-TB) each year.^1^,^2^ However, most remain undiagnosed, with high associated mortality.^3^ Children exposed to MDR-TB in their household are at high risk of developing MDR-TB,^4^ and TB preventive therapy is increasingly advised following exposure. While several clinical trials are currently underway to evaluate MDR-TB preventive therapy, evidence for safety and efficacy is currently limited.^5^ The aim of this study was to assess the feasibility and safety of a 9-month fluoroquinolone (FQ) based preventive therapy regimen in children exposed to MDR-TB in their households.

We conducted a prospective cohort study of children aged <18 years identified through a systematic MDR-TB contact investigation in the Arkhangelsk Region, Russian Federation, which has a high prevalence of MDR-TB. In 2020, MDR-TB accounted for 31% of new TB cases and 78% of retreatment cases in adults. All children consecutively identified as household contacts of confirmed pulmonary MDRTB cases, with no FQ resistance, were invited to join the study between January 2011 to March 2014. Children were followed up for at least 1 year after completion of their preventive treatment, or for 2 years if they did not receive preventive treatment. Latent TB infection (LTBI) was diagnosed based on a positive tuberculin skin test (2 tuberculin units of purified protein derivative; cut-off ≥10 mm induration) or positive Diaskintest (Generium, Moscow, Russia; culture filtrate protein 10-early secreted antigenic target 6 [CFP10-ESAT6] produced by *Escherichia coli* BL21[DE3]/pCFP-ESAT; induration of any size) in the absence of TB disease.^6^ All exposed children were offered preventive therapy with FQs irrespective of LTBI status. As per national TB guidance, all children undergoing preventive TB treatment were offered treatment in sanatoria. Children whose parents opted out from treatment in sanatoria were offered outpatient treatment. For young children, powder formulations were prepared individually for each child (using mg/kg dose) and given with food or juice. All children received either directly- or video-observed treatment by medical staff. Safety monitoring on preventive therapy included 1–4 weekly clinical reviews with medical history and 4-weekly evaluation of full blood count, alanine and aspartate aminotransferases, total bilirubin, urine analysis and ECG (with measured QT interval). Adverse drug reactions were assessed and graded according to Division of AIDS grading tables.^7^ Parents of all eligible children were invited to give informed consent for the study; parents who refused preventive treatment provided consent for the collection of routine data. Ethics approval was provided by the Northern State Medical University Ethics Committee, Arkhangelsk (no. 1; 12 January 2011).

Of 74 children identified as household contacts of MDR-TB cases, two were exposed to index cases with FQ resistance, and were therefore not eligible. Seventy-two children were included, with a median age of 7.0 years (interquartile range [IQR] 4.0–12.3; 20 (28%) were aged <5 years). All index cases were bacteriologically confirmed using culture or molecular testing (Xpert^®^ MTB/RIF, [Cepheid, Sunnyvale, CA, USA]; GenoTypeMTBDR*plus*, GenoTypeMTBDR*sl* [Hain Lifesciences, Nehren, Germany]). Sixty-three (82.9%) were sputum smear-positive for acid-fast bacilli.

In total, there were 79 index cases, with four children having household exposure to more than one MDR-TB index case. LTBI was diagnosed in 51 (71%) children (38 children had both positive TST and Diaskintest, 12 children were TST-positive only and one child was Diaskintest-positive only). There were no significant differences in children who received preventive treatment and those who did not receive treatment in terms of age at registration at TB dispensary, size of positive TB skin test reactions and duration of follow-up (Table). Fifty-eight children (81%) received preventive therapy and 52 (90%) completed the prescribed 9-month course of treatment (Table). The first three children to be treated received ofloxacin (10 mg/kg, once daily), the rest were treated with moxifloxacin (10 mg/kg, once daily), once it became available. Six children had adverse events considered to be related to the study drug. All were mild (Grade 1 or 2) and only one adverse reaction led to treatment discontinuation (allergic reaction with urticarial rash and dry cough). Fourteen children (19%) did not receive preventive therapy due to parental preference. Median follow-up was 25.1 months (IQR 18.5–30.5). Of the 58 children treated, none developed TB disease during follow-up; of 14 children who did not have preventive treatment, one child developed culture-positive TB at 18 months post-registration at the TB dispensary, with the same resistance profile as the index case.

**Table:** Baseline characteristics and management of child household contacts of MDR-TB cases in the Arkhangelsk Region, Russian Federation

	Received preventive therapy *n* (%)	Refused preventive therapy *n* (%)	Overall *n* (%)	*P* value*
Child household contacts, *n*	58	14	72	
Age at registration at TB dispensary, years, median [IQR]	7.3 [3.6–13.0]	6.0 [5.0–7.8]	7.0 [4.0–12.3]	0.44
TST induration, mm, median [IQR]	13.0 [9.5–16.0]	14.5 [10.3–16.0]	13.0 [10.0–16.0]	0.49
Diaskintest induration, mm, median [IQR]	10 [4.5–16.5]	3 [1.5–13.75]	9 [2.9–15.0]	0.37
Preventive therapy		—		
Time from registration to preventive therapy, months	1.2 [0.8–3.6]	—		
MFX	55 (95)	—		
Ofloxacin	3 (5)	—		
Duration, months, median [IQR]	9.0 [9.0–9.0]	—		
9 months	52 (90)	—		
6 months^†^	2 (3)	—		
<2 months^‡^	4 (7)	—		
Adverse reactions^§^	6 (10)	—		
Follow-up, months, median [IQR]	25.4 [18.8–30.8]	24.8 [17.9–29.4]	25.1 [18.5–30.5]	0.38
Exited study <12 months	2 (3)	0 (0)	2 (3)	
Exited study 12–24 months	2 (3)	1 (7)	3 (4)	
Transferred out	9 (16)	1 (7)	10 (14)	
LTFU	3 (5)	1 (7)	4 (6)	
Developed TB	0	1	1	N/A

* Mann-Whitney test.

^†^Two patients had 6 months of treatment because of the clinician’s decision.

^‡^Three patients interrupted treatment because of their parents’ decision, one child stopped treatment (MFX) due to an allergic reaction (urticaria and dry cough).

^§^Defined as adverse event at least possibly related to the study drug as judged by a treating clinician. Six children had adverse reactions to MFX: 2 children had Grade 1 raised ALT and/or AST at 2 and 7 months of treatment, 1 child had allergic reaction (urticaria and dry cough) of Grade 2 at 8 months of treatment, 1 child had decreased potassium of Grade 1 at 5 months and 2 had mild sinus bradyarrhythmia on ECG at 4 and 5 months of treatment.

MDR-TB = multidrug-resistant TB; IQR = interquartile range; TST = tuberculin skin test; MFX = moxifloxacin; LTFU = lost to follow-up; ALT = alanine aminotransferase; AST = aspartate aminotransferase.

Our study shows that MDR-TB preventive therapy with FQ is safe and well-tolerated by children, and none of the treated children developed TB. In 2014, TB prevention was centrally placed by the WHO as one of the main interventions in the End TB Strategy, and MDR-TB preventive therapy is currently considered to be a core element of the public health approach to MDR-TB.^5^ Our study was set up in 2011 and was among the first paediatric cohorts to explore the safety of MDR-TB preventive therapy among child household contacts. We identified a high proportion of children exposed to pulmonary MDR-TB in their household to have LTBI, and therefore at high risk of future MDR-TB disease. The study showed that FQ preventive therapy for children was feasible, and uptake was good. More than 80% of parents agreed for their child to receive preventive therapy and 90% of treated children completed the intended 9-month treatment period. Moxifloxacin was selected over levofloxacin as it was easier to access. Preventive therapy was administered under observation by medical staff, as this is routine practice in Russia. In recent years many families have opted for video-observed therapy, which eases the burden for families and health systems.

The strength of our study is a complete coverage of all identified MDR-TB child household contacts from bacteriologically confirmed pulmonary MDR-TB index cases over 3 years in the region. Despite the large catchment area, the study was limited by the modest number of exposed children, which precluded efficacy estimates. Since 2018, most international TB guidelines and TB networks recommend FQ-based preventive therapy on a case-by-case basis for household contacts of MDR-TB cases.^5^,^8^–^10^ The recommended regimen is a FQ (moxifloxacin or levofloxacin) given alone, or in combination with an additional agent to which the strain from the index case is susceptible, daily for 6–12 months. These recommendations are conditional and based on low-certainty evidence from observational and surveillance studies. Our study provides additional evidence on the feasibility and safety of FQ-based MDR-TB preventive therapy in children. Further work on the implementation of such regimens by TB programmes is urgently required.
